# Hospital Plans

**Published:** 1876-05

**Authors:** 


### Books Reviewed.

*Hospital Plans.* Five Essays relating, to the Construction, Organization and Management of Hospitals, contributed by their Authors for the use of the Johns Hopkins Hospital of Baltimore. New York: Wm. Wood & Co. 1875. Buffalo: H. H. Otis.

The late Johns Hopkins of Baltimore, made a bequest, which now amounts to three million of dollars, for the establishment and maintenance of a hospital in that city. To the trustees entrusted with the management of this fund he gives directions to secure the advice of those at home and abroad who have met with the greatest success in the construction and management of Hospitals. These gentlemen accordingly directed their building committee to confer with five distinguished physicians who had made hospitals their especial study. The gentlemen selected were Drs. John S. Billings, Asst. Surg. U. S. A.; Norton Folsom, Supt. of the Massachusetts Gen'l Hospital; Joseph Jones, of the University of Louisiana and visiting Physician to the Charity Hospital, New Orleans; Caspar Morris, late visiting Physician to the Episcopal Hospital, Philadelphia; and Stephen Smith of New York. The report of these gentleman, with the accompanying plans, and some plans and reports presented by Mr. John R. Niernsee, make up the volume.

The subjects of Hospital construction and administration are attracting much attention from the profession. They involve a discussion of some of the finer points of drainage, ventilation, architecture, etc., and are not to be decided in a moment. The gentleman who have contributed to the volume before us are all, from their experience in these matters, competent authorities. Our space will not allow a detailed mention of the various plans proposed. The gentlemen are all in favor of well built, substantial buildings, the temporary barrack style, which can be taken down and removed as desired, receiving no favor from them. The work we regard as one of great value, it contains a vast amount of information which can be found in no other work, and the trustees of the hospital deserve the thanks of the profession and the public for the publication of the truly elegant volume.

